# Perceptions of sexism interact with perceived criticism on women’s response to sexist remarks in different relationship types

**DOI:** 10.1038/s41598-023-44952-4

**Published:** 2023-10-26

**Authors:** Michelle Jin Yee Neoh, Jia Hui Teng, Peipei Setoh, Gianluca Esposito

**Affiliations:** 1 Psychology Program, School of Social Sciences, Nanyang Technological University, Singapore, Singapore; 2 Department of Psychology and Cognitive Science, University of Trento, Rovereto, Italy

**Keywords:** Psychology, Human behaviour

## Abstract

Sexism is a widespread form of gender discrimination, which can take the form of criticism towards women based on gender stereotypes. However, little is known about how perceived criticism and sexism shape one’s construal of criticism from various interpersonal sources. The present study investigated whether perceived criticism, perceived sexism and the source of criticism (mother, father, workplace supervisor, romantic partner) interact to influence upset levels in response to criticism. 178 participants completed perceived criticism (PC) ratings for the four relationships and 95 female participants also completed the Schedule of Sexist Events scale. Participants read experimental vignettes describing scenarios of criticism from different sources and rated how upset they would feel in each scenario. Perceived sexism significantly moderated the effect of PC on upset levels only for sexist-related criticism from romantic partners and supervisors. Female participants with low perceived sexism show higher levels of upset as PC increased for sexist-related criticism from supervisors whereas female participants with high perceived sexism show lower levels of upset as PC increased for sexist-related criticism from romantic partners. These findings contribute towards understanding how perceived criticism and perceived sexism influence affective reactions to criticism across interpersonal sources.

## Introduction

A normal and integral part of performance feedback, criticism can be defined as the negative evaluation of an individual that is articulated by another^1^, which may include expressions of dissatisfaction. Negative social evaluation often leads to negative emotional responses, such as anger and sadness^2^, as critical comments are characterized by emotional arousal and personal relevance^3^. Criticism can also be destructive when perceived to be harmful or hurtful. Criticism may be construed as a source of threat to one’s ego and face, and lead to emotional distress and defensiveness^4^. It has also been recognized as a robust predictor of clinical outcomes, including anxiety disorders such as panic disorder and mood disorders such as major depressive disorder^5^. Criticism is prevalent in gender discrimination, such as sexism, which is a multifaceted construct that encompasses hostility and prejudice against women^6^. Sexism can be conceptualized as consisting of two complementary dimensions encompassing both hostile and benevolent attitudes toward women according to ambivalent sexism theory^6^. Hostile sexism involves overt antipathy and negative stereotypes about women including beliefs that women are incompetent, overly emotional and manipulative whereas benevolent sexism involves “positive” views of women consisting of well-intentioned and paternalistic attitudes and behaviours towards women such as portrayals of women as “pure”, idealized caregivers and needing protection^6^. Given that sexist remarks can be construed as critical by the recipient, the present study investigated the influence of perceived criticism on the intensity of negative emotional responses, specifically upset levels, towards criticism originating from different sources in both public and private domains, and how perceived sexism may moderate upset levels.

## Criticism in interpersonal relationships

The way individuals perceive and cognitively process criticism is dependent on the relational and institutional contexts that the individual is situated in^1^. In other words, the same statement articulated within a different relational context can be perceived differently by the same individual. For example, a neuroimaging study found that individuals with high PC ratings of their mothers experienced an increased activation in the DLPFC when reading vignettes depicting criticism from parents and romantic partners from a third-party perspective, but a decreased activation for criticism from friends, compared to individuals with low PC ratings^7^. Moreover, participants were more likely to remember feedback from close others such as romantic partners and friends but were less likely to remember feedback received from strangers^8^. Another relational context where criticism plays an integral role is the organisational setting as employees receive performance evaluations and feedback from supervisors. Empirical studies have found that employees’ satisfaction with supervisors is highly correlated to performance appraisal satisfaction^9,10^. Employees who have a more trusting and amiable relationship with their supervisors reported more positive reactions to feedback, unlike those who have impersonal and less favorable relationships^11^. Taken together, it can be expected that criticism from different sources will elicit different intensities of emotional reactions given the difference in the nature of close and work relationships.

### Perceived criticism

One factor that can influence the response to criticism is an individual’s perception of the criticism. Perceived criticism (PC) is a subjective measure of criticism perceived by a recipient in a meaningful and emotionally important relationship^5^, such as with parents, spouse or partner. PC was developed as a simplified measurement of the expressed emotion construct, where criticism was the element with the most consistent links with poor clinical outcomes^12^. For example, high expressed emotion spouses were more negative and less positive towards their partners, where they made more critical remarks and disagreed more frequently with their partners^13^. In addition, individuals living in family environments characterized by high levels of criticism were more likely to experience relapses in depression^14^. PC was found to be negatively associated with relationship satisfaction^15^, and higher PC predicts poorer clinical outcomes transdiagnostically and relapse rates over a myriad of clinical disorders^5^, highlighting the importance of studying PC and its negative consequences on individuals. PC is positively associated with perceptions of destructive criticism^16^, and is a representation of the amount of criticism that gets internalized by an individual^17^. This could be contributed by actual criticism that an individual receives from a close other, wherein the received criticism trains the individual to be more reactive and hypersensitive to negative emotional cues as they become more salient^8^. In addition, critical comments have been found to be perceived to be arousing and personal relevant (e.g.,^18,19^), where personal relevance of criticism was found to show a significant positive correlation with PC ratings as well as negative affect^3^. This may result in increased criticality or interpretation biases, which is the tendency to over-perceive criticism directed at the recipient than is actually intended or present^20^. Similarly, rejection sensitivity, which is the disposition of expecting, readily perceiving, and overreacting to rejection^21^, is also related to perceptions of rejection. Individuals high on rejection sensitivity tend to perceive criticism and hostility or perceive intentional rejection even in the absence of such intentions. Studies have found that individuals who were high in rejection sensitivity reported higher anxiety and avoidance in relationships and were more negative in interactions with romantic partners^22^. Moreover, the relationships of individuals who were high in rejection sensitivity were observed to be more likely to dissolve^23^. Such biases suggest that PC ratings for a relational partner can reflect how individuals perceive and respond to comments made in their relationships with others. Hence, how critical an individual perceives the relational partner to be can influence their perceptions of criticism from a relational partner and their consequent emotional response to these comments.

Previous studies have investigated the link between PC and upset experienced in response to criticism. PC ratings for a parent or romantic partner were found to be significantly correlated with participants’ ratings of how upset they become in response to criticism (e.g.,^24,25^). Similarly, studies have also reported findings which indicate the relationship between perceived criticism with negative affect. For example, significant correlations have been reported between PC ratings and self-reported depressive symptom scores (e.g.,^26^), where such correlations were observed in individuals with a history of substance use^27^. Additionally, negative affect and perceived irritability were found to fully mediate the effect of PC ratings on positive schizotypy, indicating a relationship between PC and negative affect (e.g.,^3^). Hence, it can be expected that perceptions of how critical a relative is can be predictive of the emotional response experienced in response to criticism received from these relatives.

## Experiences of sexism

In almost every society throughout history, women have occupied a lower social status than men and are more often targets of discrimination^28,29^. A number of studies have shown that sexist interpersonal interactions; including sexual harassment, unfair treatment or being discriminated against on the basis of their gender, are relatively frequent and impactful occurrences in the daily lives of women (e.g.,^30,31^). For example, female undergraduates reported one to two impactful sexist incidents a week^32^ and examples of everyday sexism directed at women and girls were described extensively by secondary school students whereas very few instances of “sexism” towards men and boys were described^33^. Klonoff and Landrine’s^34^ study found that women of various socio-economic statuses, education levels, age, marital statuses, and cultures may have different socialisation experiences, but had all been discriminated against for being a woman.

With regards to sexism, sexist events can be perpetrated by a variety of sources^32^. Sexist beliefs, such as those about the behaviours of men, also differ across relationships where perceptions of events as sexist were found to differ according to the perpetrator of sexism^35,36^. Specifically, comments made by a boyfriend were rated as less sexist than when these same comments were made by a boss or stranger^35^. Parents are primary socialising agents of children where parents are commonly cited as primary sources of sexism^37^. Parents can articulate, impose and reinforce their conceptions of gender and gender discrimination onto their children across their upbringing^38^. Parents are the first models of behaviors related to social and gender roles^39^, where parental attitudes have been argued to be one of the main influences of parents on children^40^. Parental expectations and attitudes about gender and gender roles shape their interactions, communication, and behaviours with their children^41^. For example, gendered parenting—messages received by children from parents regarding behaviours of boys and girls^42^—can include explicit encouragement of sex-typed activities^38^ and parents’ choices of colours, toys, clothing and room decoration can also reinforce gendered behaviour^43^. A recent review by Morawska^44^ reported evidence of differential parenting of sons and daughters such as socialising strategies and vocalisations, which were found to be associated with differences in child behaviors such as displays of affect, toy play, and aggression. Children exposed to greater sexist beliefs from their parents may cultivate greater sexist attitudes and develop a greater immunity and ability to accept sexism, therefore perceiving less sexism. The workplace is another institutional setting where sexism can occur. Women still face pervasive gender stereotypes and discrimination in the workplace^45^, including negative stereotypes that women have lower leadership ability, career commitment and emotional stability^46,47^. Women also encounter gender harassment, which consists of a range of verbal and non-verbal behaviours conveying sexist, insulting or hostile attitudes about women including negative comments about women, sexist jokes and sexist behaviour.

Sexist remarks being made against women, which often involve negative stereotypes about women and/or involve negative attitudes and evaluations about various subject matters such as appearance, abilities, professionalism and traits and behaviours, are a common sexist interaction. The most commonly experienced emotions in response to sexist events including stereotypes and derogatory comments or behaviours was anger and upset^32^. Such negative emotional responses are commonly reported in response to experiences of sexism across studies including feelings of anger, weakness, tenseness, sadness^48–51^. Research has demonstrated that women are more readily able to identify hostile sexist comments compared to benevolent comments^6^ and hostile sexism events were perceived to be more distressing and sexist^30^. Since hostile sexism is the overt prejudice and devaluation of women, sexist comments involving elements of hostile sexism such as negative stereotypes would likely be construed as criticism when negative evaluations are involved. For example, a study conducted by the Utah Women & Leadership Project reported that a majority of sexist remarks involving gender stereotypes tended to involve negative generalisations about women, such as “Women were too irrational and emotional to be good legislators”^52^, which can be construed as criticism against the emotionality and abilities of women. The expression of negative evaluations about women in such sexist remarks can be construed as destructive criticism by the women receiving them, especially when the remarks are derogatory and demeaning. Such remarks can cause individuals to feel upset, insulted, humiliated, or experience emotional and cognitive consequences akin to when they receive criticism. Hence, perceptions of sexism may influence perceptions and processing of criticism, especially critical comments involving elements of sexism.

### Perceived sexism

Perceived sexism is associated with negative emotional and psychological consequences such as obsessive-compulsivity and anxiety^53^. Within groups that are often ostracized and stigmatized, such as women and ethnic minorities, recognizing that one’s ingroup is a target of prejudice and discrimination is negatively related to emotional well-being and self-esteem levels^54^ since an individual’s perception and feelings of worth is, in part, reliant on their group membership. When individuals perceive their social group to be targets of pervasive discrimination, expectations of discrimination become more likely, leading to expectations of future negative treatment across different social contexts (see^55^). In accordance with social identity theory, Schmitt and Branscombe^51^ propose that since the social identity is a cornerstone of the self, prejudice against the individual’s ingroup will be perceived not only as a threat to the group and social identity, but also a threat to the self. This is supported by their findings where rejection by a sexist professor resulted in greater negative affect and internal causal attributions among females than rejection by a professor who rejected everyone regardless of gender. Similarly, Pinel^56^ proposed that there are individual differences in stigma consciousness—the extent to which targets believe that their stereotyped status pervades interactions with outgroup members. In other words, some individuals may be more sensitive or conscious of being the target of stereotyping, prejudice and discrimination where individuals high in stigma consciousness are more likely to perceive discrimination. For example, a higher likelihood of attributing ambiguous negative feedback from men to sexism was observed in women high in trait stigma consciousness (i.e., those scoring in the upper third of a pre-screening distribution of the Stigma Consciousness Questionnaire^56^). Although women low in trait stigma consciousness (i.e., those scoring in the lower third of a pre-screening distribution of the Stigma Consciousness Questionnaire) were found to demonstrate a lower tendency to make attributions to discrimination regarding feedback, they showed as great a tendency as high stigma conscious women when they experienced a situationally induced increase in stigma consciousness^57^. Stangor et al.^58^ found that women who indicated a higher frequency of sexist experiences by themselves and others reported having seen more news headlines regarding sexism than was actually presented whereas low and medium-sensitive women underestimated this number. Similarly, gender stigma consciousness was found to predict impostor phenomenon in both male and female undergraduates, where the effect was observed to be stronger in women^59^. Hence, we expect that individuals who perceive a higher frequency of sexist experiences would perceive greater sexism in everyday life and experience greater negative affect towards sexist events such as sexist-related criticism across different relational contexts since such experiences cultivate greater sensitivity and consciousness of sexism.

## Present study

Both criticism and sexism occur across a range of relationships, where the relational context in which a particular event occurs in influences whether it is perceived as criticism and/or sexism by the individual. Since studies examining the effects of criticism from multiple interpersonal sources are scarce, this study will contribute to the current literature by comparing the effects of criticism in four different relational contexts—mother, father, workplace supervisor and romantic partner. Sexism is also commonly perpetrated in these relationships. Investigating these relationships can also provide insight into possible differences between close and work relationships, both of which are significant constituents of an individual’s social environment.

To the best of our knowledge, perceived criticism and perceived sexism have not been examined as possible factors influencing the affective interpretation and construal of critical feedback in social interactions across different interpersonal sources. There have also not been any studies examining the relationship between an individual’s perceptions of criticism and perceptions of sexism on the negative emotional reactions towards sexist-related criticism from different sources. Given that sexist remarks are prevalent across both private and public spheres and appear to be normalized across most social settings, including workplaces, families, schools, and communities^52^, it is important to understand how perceptions of criticism and sexism influence emotional responses towards sexist-related criticism. Such sexist experiences in everyday can have repercussions on psychological well-being of women, where perceived discrimination and discrimination based on gender have been shown to be negatively associated with psychological well-being (e.g.,^29,60,61^). Moreover, the perceptions and emotional responses towards experiences of sexism such as sexist remarks have implications on how individuals respond towards these experiences, and consequently, the perpetuation of such stereotypes and behaviors (see^62^). If the remark is not perceived as sexist, then it is unlikely to be accompanied by negative emotional responses or confrontation. When sexism is not confronted, this implies the behavior is appropriate and condoned^63^. Specifically, perpetrators of sexism are made aware of their inappropriate behavior and become wary of reoffending when confronted^64,65^. These results indicate that how sexist incidents are responded to can contribute to the perpetuation of such sexist attitudes and behaviors, highlighting the importance of understanding the factors that influence one’s responses to such events. In addition, although some studies have reported findings indicating differences in the perceptions of sexist behaviors from different perpetrators (e.g.,^35,66^), a majority of existing studies investigating perceptions and responses to sexism did not consider the relational context in which the sexist incident took place in and were often (i) in the context of work settings (e.g.,^67,68^) or (ii) the “male interaction partner” in the scenarios were strangers (e.g.,^69,70^).

Additionally, the majority of studies on PC have been conducted in North American and European samples and few studies have investigated the relationship between PC and upset by criticism in Asian contexts (e.g.,^71^). Cultural differences may play a role in the relationship between PC and upset by criticism where differences in upset by perceived criticism between Black and White participants were found previously^25^. Given differences in levels of criticism across cultures (e.g.,^72,73^), this study can contribute to the study of PC in different cultural contexts.

Hence, the present study aims to investigate the negative emotional responses towards criticism encountered in familial, workplace and intimate interactions and the possible moderating effects of perceived criticism and perceived sexism. Consequently, the findings from the present study can contribute to existing literature by opening up a new line of inquiry in how sexism may permeate everyday social interactions and how perceptions of criticism and sexism may possibly play a role in how individuals perceive and react to criticism.

We expect that there will be differences in upset experienced when individuals are exposed to hypothetical scenarios that describe criticism received from parents, workplace supervisors and romantic partners in a first-person perspective. Since perceived criticism depends on the context of the relationship, we expect that there are differences in levels of upset between individuals with higher levels of perceived criticism compared to those with lower levels of perceived criticism for different vignettes involving criticism occurring in different relationship types. Our hypotheses are as follows:

### Hypothesis 1

There is a significant interaction between perceived criticism and relationship type on the level of upset experienced in response to criticism. Specifically, we expect that with higher perceived criticism ratings for workplace supervisors, greater levels of upset will be experienced in response to criticism. Such an increase in negative emotional response to criticism as perceived criticism ratings increase will not be expected to be observed for close relationships (i.e., mothers, fathers and romantic partners).

Additionally, since perceived sexism considerably influences how people perceive, construe and respond to sexist-related criticism, there is a potential association among perceived criticism, relationship type and perceived sexism on upset levels experienced. Only the female sample was examined as females are more likely to be targets of sexist acts compared to their male counterparts in their everyday lives^74^. Specifically, we hypothesised that in the female sample, there will be different levels of upset for different relationship types based on perceived criticism ratings in individuals with high versus low perceived sexism.

### Hypothesis 2

There is a significant three-way interaction effect in the relationship among perceived criticism, relationship type, and perceived sexism on the level of upset experienced by female participants.

Based on previous results from Riemer et al.^35^, we expect that female participants with higher perceived experiences of sexism will be more likely to perceive sexist-related criticism from supervisors as sexist compared to when it originates from a romantic partner. Moreover, previous results have indicated that motivations for relationship maintenance and closeness can bias perceptions of partners (see^75,76^), where such tendencies for motivated cognition are more likely to be observed in the romantic relationship than a working relationship. Hence, we expect that female participants with high perceived sexism will experience significantly greater levels of upset in response to sexist-related criticism when PC ratings increase compared to female participants with low perceived sexism for sexist-related criticism from supervisors, but not for romantic partners.

## Methodology

### Participants

A total of 178 (female = 95, male = 83) participants were recruited. Participants were recruited through (i) word-of-mouth compensated with remuneration and (ii) psychology undergraduates compensated with course credits. Majority of the participants were university students (*n* = 146). Participants of 18 to 35 years of age and having no disabilities or difficulties with English were the inclusion criteria for the study. The method and procedure of this study was approved by the Psychology Ethics Committee at Nanyang Technological University (PSY-IRB-2020–007). The study was carried out in accordance with the Declaration of Helsinki. Written informed consent was obtained from participants prior to the commencement of the study.

### Experimental questionnaires

Participants first completed a set of online questionnaires hosted on Qualtrics that included demographic questions and the following scales.

#### Perceived criticism measure

An item from the Perceived Criticism Measure^77^ that asks about individuals’ perceptions of others being critical of them (e.g., “How critical do you think your mother is of you?”) was used. Measured on a 10-point Likert scale from 1 *(not at all critical)* to 10 *(very critical)*, the Perceived Criticism Measure has demonstrated good test–retest reliability, construct and predictive validity, as well as positive correlation with expressed emotions^15,78^.

#### Schedule of sexist events (SSE)

The SSE-Lifetime is a 20-item self-report questionnaire that assesses the frequency of gender discrimination that a woman experiences in her lifetime^34^. Female participants rated on a 6-point Likert-type scale, ranging from 1 (“*the event never happened”*) to 6 (“*the event happens almost all of the time*”). The SSE-Lifetime has high internal reliability, and convergent validity with other well-known measures of stress such as the PERI Life Events Scale^34^. The Cronbach’s alpha for the SSE in this study was α = 0.93.

#### Relationship quality

Relationship quality was measured using questions adapted from the Quality of Marriage Index (QMI)^79^. There were 6 items where 5 items were rated on a 7-point Likert scale and 1 item rated was on a 10-point Likert scale, with higher scores reflecting better relationship quality.

### Experimental stimuli

After completing the online questionnaires, participants were asked to read and answer questions regarding experimental vignettes, which were hosted on a separate Qualtrics link. The vignettes depicted four scenarios involving comments regarding commonplace themes of intelligence, empathy, accountability and assertiveness. Two of the scenarios (intelligence, assertiveness) involved elements of sexism in the comments being made. There are two versions of each of the four scenarios; a praise and criticism condition, hence participants read eight experimental vignettes in total. While the four scenarios were presented in the same order, participants were randomly assigned to different groups, where the order of the relationship type described in each vignette was different. The order of the relationship types being described in the vignettes are counterbalanced as follows: (a) Mother-Father-Partner-Supervisor, (b) Father-Supervisor-Mother-Partner, (c) Supervisor-Partner-Father-Mother and (d) Partner-Mother-Supervisor-Father. Vignettes presented to male and female participants also had slight variations. The versions for each gender differed in gender pronouns used, and content of the vignettes alluded to prevailing gender stereotypes. For example, the comment for the sexist praise condition for female participants was “Well done! Girls usually excel in Languages, and I am glad you did well. Keep up the good work. If you continue to work hard and make unremitting efforts towards your studies, you will be successful in your future. Keep up the good work and this mindset.” An example of the comment for the sexist criticism condition for male participants was “Most guys can be too dominant and aggressive in asserting their opinions when others have a different opinion from theirs. And you are just like them. You want things to be done your way without listening to others nor are you willing to compromise. You should listen to others’ opinions and not force others to accept yours. You should change that pushy attitude of yours.”

Each vignette contained approximately 120 words, and followed a similar structure, whereby a situational context is first delineated, followed by a block quote that conveys either praise or criticism by the source. To ensure participants report the intensity of emotional responses experienced in relation to the vignettes as accurately as possible, the vignettes were written in the first-person perspective to heighten identification with the protagonist. After reading each vignette, participants were asked to imagine that they were in the same situation described in the vignette and rate how upset they would feel upon receiving the criticism on a 5-point scale (with 1 being *not at all* and 5 being *completely*).

### Statistical analyses

Data collected from the online questionnaires were analysed using IBM SPSS Version 25.0 (IBM Corps, Armonk, NY, USA) and RStudio Version 1.3 software. Descriptive statistics such as the frequencies and central tendency measurements were calculated for demographic data. Moderation analysis were then conducted to study (i) the relationship between PC and relationship type on upset, and (ii) the relationship among PC, perceived sexism, relationship types on upset experienced. The dataset analysed in this study is available in the open access institutional data repository at the link: https://doi.org/10.21979/N9/APYGCF.

To test Hypothesis 1, multiple regression was conducted on the ratings of upset experienced after reading the experimental vignettes. A model was fitted for each gender for each comment type (i) sexist-related criticism and (ii) non-sexist criticism. The following predictors were included in the model: (i) PC ratings, (ii) relationship type (4 levels: Father, Mother, Partner, and Supervisor), and (iii) the interaction term PC * Relationship type. Since relationship type is a categorical predictor, it was dummy coded with 3 dummy variables. The significance of the interaction term was tested using model comparison (i.e., the model containing the interaction term versus the model without the interaction term). Relationship quality was also included in the model as a covariate. The means and standard deviations of levels of upset and PC by gender and relationship type are presented in Table 1.

**Table 1:** Means and standard deviations of levels of upset grouped by gender for each comment type.

	PC Mean (SD)	Sexist criticism		Non-sexist criticism	
		M	SD	M	SD
Male					
Mother	4.82 (3.14)	3.51	0.97	2.80	1.30
Father	4.30 (2.78)	3.47	1.16	2.86	1.25
Supervisor	4.80 (2.79)	3.63	1.02	3.21	1.24
Romantic partner	4.17 (2.73)	3.79	1.17	3.40	1.14
Female					
Mother	5.25 (2.64)	4.04	0.97	3.30	1.20
Father	4.76 (2.83)	4.17	0.94	3.42	1.23
Supervisor	4.67 (2.46)	4.07	1.12	3.48	1.28
Romantic partner	4.41 (2.84)	3.92	1.20	3.26	1.18

To test Hypothesis 2 which looks at the effects of perceived sexist events in the female sample, multiple regression was conducted on the ratings of upset experienced after reading the experimental vignettes. A model was fitted for each comment type (i) sexist-related criticism and (ii) non-sexist criticism. The following predictors were included in the model: (i) PC ratings, (ii) relationship type (4 levels: Father, Mother, Partner, and Supervisor), (iii) SSE ratings, (iv) the two-way interactions (PC * Relationship type, PC * SSE, Relationship type * SSE), and (v) the three-way interaction PC * Relationship type * SSE. Since relationship type is a categorical predictor, it was dummy coded with 3 dummy variables. The significance of the interaction term was tested using model comparison (i.e., the model containing the interaction term versus the model without the interaction term). Relationship quality was also included in the model as a covariate. The means and SDs of levels of upset for each comment type are presented in Table 2.

**Table 2:** Means and standard deviations of levels of upset for each relationship type grouped by perceived sexism towards each comment type.

	PC Mean (SD)	Sexist criticism		Non-sexist criticism	
		M	SD	M	SD
High perceived sexism					
Mother	5.52 (2.46)	4.09	0.85	3.48	1.09
Father	5.42 (2.80)	4.30	0.78	3.35	0.93
Supervisor	4.49 (2.39)	4.14	0.89	3.68	1.25
Romantic partner	4.44 (2.93)	3.94	1.29	3.09	1.04
Low perceived sexism					
Mother	4.96 (2.84)	4.00	1.08	3.05	1.32
Father	4.02 (2.72)	4.00	1.12	3.48	1.48
Supervisor	4.86 (2.56)	4.00	1.32	3.21	1.32
Romantic partner	4.36 (2.80)	3.90	1.10	3.42	1.31

To facilitate comparison in this table, high (*n* = 50) and low (*n* = 45) perceived sexism were grouped by a median split of SSE scores (median = 39). Mean SSE scores in the sample = 42.7.

## Results

### Descriptive statistics

The age of participants ranged from 18 to 31 years. The average age of participants was 21.3 years (*SD* = 2.23), where *M*_*male*_ = 22.0, *SD*_*male*_ = 2.12, and *M*_*female*_ = 20.7, *SD*_*female*_ = 2.12. The sample was made up of Chinese (*n* = 135, 75.8%), Malay (*n* = 8, 4.5%), Indian (*n* = 13, 7.3%) and other races (*n* = 15, 8.4%). 170 participants (female = 90) are currently working or have working experience. 47 participants (female = 24) are currently in a romantic relationship, 50 participants (female = 25) were previously but are not currently in a romantic relationship and the remaining 81 participants (female = 46) had never been in a romantic relationship before. Ratings for the vignettes involving criticism from (i) romantic partners and (ii) supervisors respectively were not included in the data analysis for participants who indicated that they (i) have never been in a romantic relationship and/or (ii) did not have previous work experience.

### Preliminary analyses

The correlation between ratings of upset with PC ratings was not significant in both the male (*r* = 0.002, *p* = 0.97) and female sample (*r* = − 0.01, *p* = 0.81). The correlation between ratings of upset with QMI ratings was also not significant in both the male (*r* = − 0.03, *p* = 0.58) and female sample (*r* = 0.08, *p* = 0.17). In the female sample, the correlation between SSE scores and PC ratings was significant (*r* = 0.11, *p* = 0.048). The correlations between Schedule of Sexist Events with (i) ratings of upset (*r* = 0.03, *p* = 0.59) and (ii) QMI ratings were not significant (*r* = 0.01, *p* = 0.83). PC ratings ranged from 1 to 10 (*M* = 4.68, SD = 2.71) and the range of observed values of SSE scores in this sample was 22 to 88 (*M* = 42.70, SD = 15.86).

### Main analyses

#### Interaction of relationship type and PC

**Figure 1:**
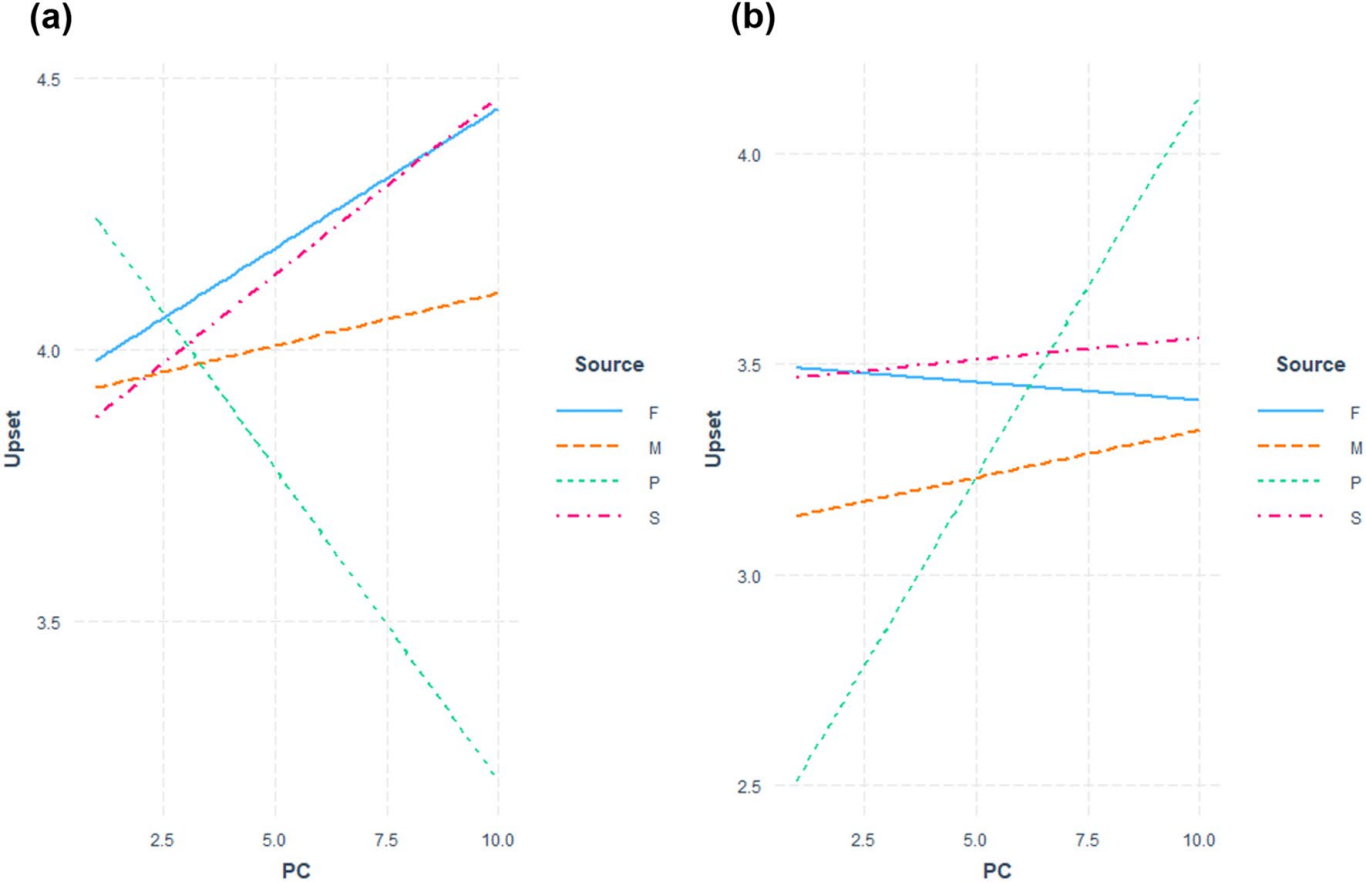
Interaction plots of fitted regression lines of PC against levels of upset grouped by relationship type (F: Father, M: Mother, P: Partner, S: Supervisor) for the female group (A: sexist comments, B: nonsexist comments).

**Figure 2:**
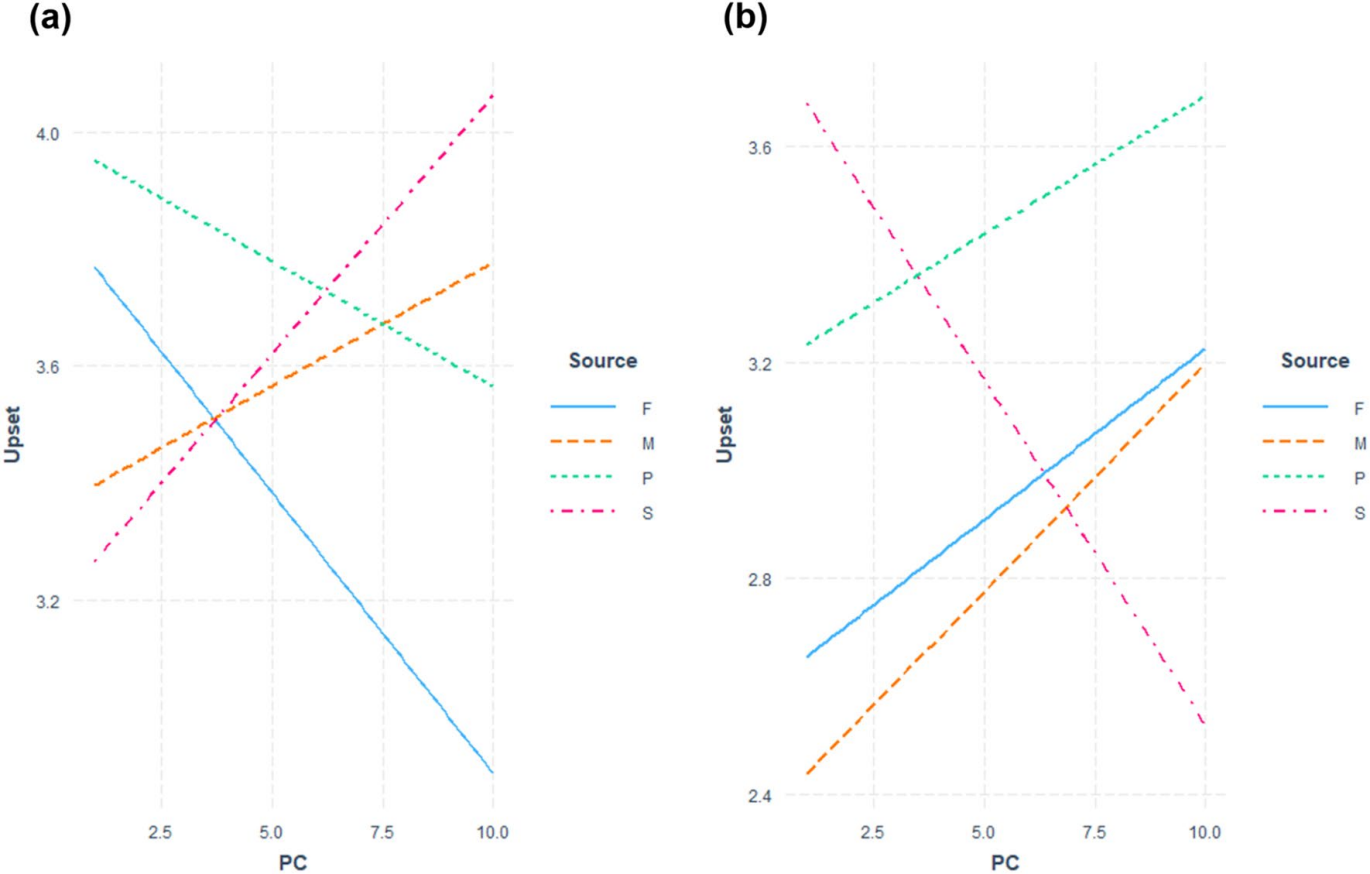
Interaction plots of fitted regression lines of PC against levels of upset grouped by relationship type (F: Father, M: Mother, P: Partner, S: Supervisor) for the male group (A: sexist comments, B: nonsexist comments).

The fitted regression model to test Hypothesis 1 is summarized in Table 3 and 4. Figures 1 and 2 show the regression plots for the female and male groups respectively. With regards to sexist comments, model comparison indicated that the interaction effect of relationship type x PC was not significant in both the male group (F(3, 143) = 1.81, *p* = 0.15) and the female group (F(3, 158) = 1.62, *p* = 0.19). In the male group, the coefficient for the interaction term PC * Source (Supervisor) was significant (*t* = 2.13, *p* = 0.04). PC and relationship type were not significant predictors in the regression model for the female group.

**Table 3:** Regression model for upset predicted by relationship type and PC for the female group.

Predictor	Sexist criticism			Non-sexist criticism		
	β	*t*-value	*p*-value	β	*t*-value	*p*-value
PC	0.05	0.94	.35	− 0.01	− 0.13	.90
Source (Mother)	− 0.02	− 0.04	.97	− 0.38	− 0.73	.47
Source (partner)	0.43	1.00	.32	− 1.17	− 1.59	.11
Source (supervisor)	− 0.12	− 0.27	.79	− 0.04	− 0.08	.94
Relationship quality	0.01	0.89	.38	0.02	1.48	.14
PC * source (mother)	− 0.03	− 0.40	.69	0.03	0.34	.73
PC * source (partner)	− 0.17	− 1.96	.05	0.19	1.45	.15
PC * source (supervisor)	0.01	0.16	.87	0.02	0.20	.85
	R^2^ = 0.04			R^2^ = 0.03		

PC: Perceived criticism.

Source was dummy coded with Father: 0.

**Table 4:** Regression model for upset predicted by relationship type and PC for the male group.

Predictor	Sexist criticism			Non-sexist criticism		
	β	*t*-value	*p*-value	β	*t*-value	*p*-value
PC	− 0.10	− 1.64	.10	0.06	0.87	.38
Source (mother)	− 0.51	− 1.22	.22	− 0.24	− 0.45	.66
Source (partner)	0.60	0.27	.79	0.59	0.94	.35
Source (supervisor)	− 0.69	− 1.48	.14	1.22	2.29	.02*
Relationship quality	− 0.007	− 0.77	.44	− 0.004	− 0.378	.71
PC * source (mother)	0.14	1.69	.09	0.02	0.22	.82
PC * source (partner)	0.05	0.54	.59	− 0.01	− 0.10	.92
PC * source (supervisor)	0.18	2.13	.04*	− 0.19	− 1.92	.05
	R^2^ = 0.05			R^2^ = 0.08		

PC: Perceived criticism.

*p < .05.

Source was dummy coded with Father: 0.

With regards to nonsexist comments, model comparison indicated that the interaction effect of relationship type x PC was not significant in both male (F(3, 133) = 2.09, *p* = 0.10) and female (F(3, 153) = 0.73, *p* = 0.53) groups for nonsexist comments. PC and relationship type were also not significant predictors in the regression model for the female group. However, there was a significant main effect of relationship type in the male group where the regression coefficient for Supervisor was significant (*t* = 2.29, *p* = 0.02) (Table 4).

#### Interaction of relationship type, PC and perceived sexism

**Figure 3:**
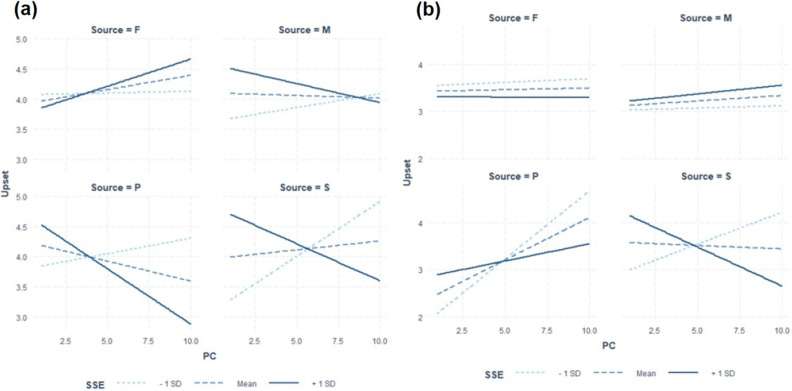
Interaction plots of fitted regression lines of PC and perceived sexism against levels of upset grouped by relationship type (F: Father, M: Mother, P: Partner, S: Supervisor) (A: sexist comments, B: nonsexist comments). *Note.* Slopes are graphed at (i) 1 standard deviation above (+ 1 SD), (ii) below (- 1 SD) and (iii) at the mean of the scores on the SSE.

**Figure 4:**
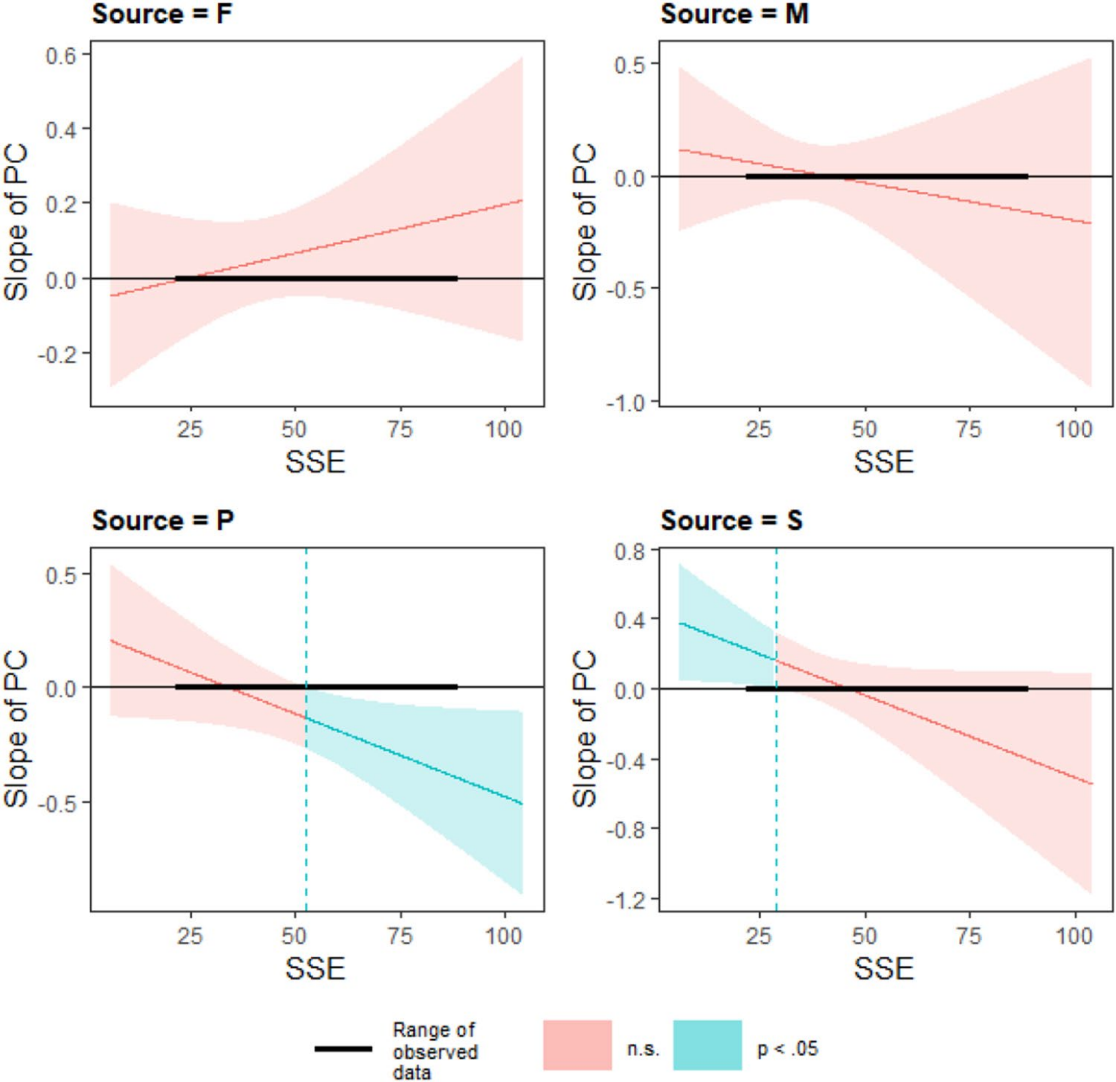
Plots of slopes of PC against SSE scores for each relationship type (F: Father, M: Mother, P: Partner, S: Supervisor) for sexist comments. *Note*. The Johnson-Neyman interval when the slope of PC is significant is indicated by the region in blue.

The fitted regression models to test Hypothesis 2 are summarized in Table 5 and Fig. 3 shows the regression plots. With regards to sexist comments, model comparison indicated that the three-way interaction effect of relationship type x PC x perceived sexism was close to significance (F(3, 153) = 2.23, *p* = 0.088), where the regression coefficients for the three-way interaction PC * SSE * Source (Partner) (*t* = − 2.11, *p* = 0.04) and PC * SSE * Source (Supervisor) (*t* = − 2.13, *p* = 0.04) were significant (Table 5). Simple slopes analysis indicated that a two-way interaction between PC and perceived sexism was only significant for romantic partners and supervisors. The Johnson-Neyman technique^80^ was used to identify the values when the slope of PC was significant (Fig. 4). For romantic partners, the slope of PC was significant for female participants who scored higher on the SSE (high perceived sexism; mean + 1 SD) (*t* = − 2.43, *p* = 0.02). The slope of PC was significant for values above 52.52. The moderation was such that levels of upset decreased as PC ratings for romantic partners increased for female participants with high perceived sexism. For supervisors, the slope of PC was significant for female participants who scored lower on the SSE (low perceived sexism; mean—1 SD) (*t* = 2.06, *p* = 0.02). The slope of PC was significant for values below 28.67. The moderation was such that levels of upset increased as PC ratings for supervisors increased for female participants with low perceived sexism. Hence, findings in the study did not support Hypothesis 2.

**Table 5:** Regression model for upset predicted by relationship type, PC and perceived sexism for female participants (N = 95).

Predictor	Sexist criticism			Non-sexist criticism		
	β	*t*-value	*p*-value	β	*t*-value	*p*-value
Relationship quality	0.01	1.01	.31	0.02	1.59	.11
PC	− 0.06	− 0.45	.65	0.03	0.16	.87
SSE	− 0.01	− 0.58	.57	− 0.01	− 0.32	.75
PC * SSE	0.002	0.86	.39	− 0.0005	− 0.14	.89
Source (mother)	− 1.47	− 0.96	.34	− 0.85	− 0.62	.54
Source (partner)	− 1.29	− 1.16	.25	− 2.85	− 1.03	.31
Source (supervisor)	− 2.65	− 2.15	.03*	− 2.12	− 1.36	.18
PC * source (mother)	0.20	0.78	.44	− 0.04	− 0.17	.86
PC * source (partner)	0.31	1.31	.19	0.44	1.00	.32
PC * source (supervisor)	0.50	2.06	.04*	0.37	1.23	.22
SSE * source (mother)	0.04	0.98	.33	0.012	0.40	.69
SSE * source (partner)	0.04	1.56	.12	0.04	0.67	.50
SSE * source (supervisor)	0.06	2.18	.03*	0.05	1.48	.14
PC * SSE * source (mother)	− 0.01	− 0.96	.34	0.001	0.25	.80
PC * SSE *source (partner)	− 0.01	− 2.11	.04*	− 0.006	− 0.65	.51
PC * SSE * source (supervisor)	− 0.01	− 2.13	.04*	− 0.009	− 1.33	.18
		R^2^ = 0.11			R^2^ = 0.07	

PC: Perceived Criticism as measured with PC ratings, SSE: Schedule of Sexist Events ratings.

*p < .05.

On the other hand, with regards to nonsexist comments, model comparison indicated that the three-way interaction effect of relationship type x PC x perceived sexism was not significant (F(3, 148) = 0.95, *p* = 0.42) (Fig. 3). The two-way interaction terms, PC, relationship type and perceived sexism were also not significant predictors in the model.

## Discussion

A significant interaction effect of relationship type x PC was found in the male group for sexist-related criticism but not in the female group. As PC ratings for supervisors increased, the levels of upset experienced increased. However, this association between PC ratings and levels of upset was not observed for the other relationship types (mother, father, romantic partner). Hence, Hypothesis 1 was supported, suggesting that negative affect in response to sexist-related criticism may vary according to the relationship type in the male group. This finding also aligns with Hooley et al.’s finding that higher perceptions of maternal criticism were not associated with an increased negative emotional response or greater upset^81^.

A significant three-way interaction of relationship type x PC x perceived sexism was found in the female group for sexist-related criticism. The three-way interaction was such that perceived sexism significantly moderated the effect of PC on levels of upset only for sexist-related criticism from romantic partners and supervisors. Firstly, the levels of upset experienced increased as PC ratings for supervisors increased only for female participants with low perceived sexism. This finding may be due to differences in the attributions made about a supervisor's comments. Comments from bosses have previously been found to be more sexist compared to those from a romantic partner^35^, suggesting that individuals with high perceived sexism will be more likely to identify sexist-related criticism as sexism on the part of the supervisor. Consequently, this may make criticism and sexist remarks easier to brush off and attributing it to external sources such as the supervisor’s disposition or sexism by the supervisor rather than internalising it, thereby being less affected emotionally. On the other hand, individuals with low perceived sexism may be less likely to explain the sexist-related criticism as a sexist incident and make negative attributions relating to themselves about the criticism, resulting in greater feelings of upset. As a result, the more supervisors are perceived to be more critical, individuals with high perceived sexism could be more likely to attribute sexist-related criticism to the supervisor’s sexism whereas individuals with low perceived sexism may be less likely to attribute sexist-related to sexism by a supervisor and internalize sexist-related criticism as personal shortcomings, leading to greater upset being experienced. Another possible explanation could be that individuals with high perceived sexism are more “habituated” to sexist experiences and have a reduced emotional response when encountering sexist-related criticism whereas individuals with low perceived sexism are more prone to negative emotional responses towards sexist incidents. As a result, female participants with low perceived sexism who perceived their supervisors as more critical of them may be more likely to have a greater negative emotional response towards the sexist-related criticism. For example, interaction styles in families appeared to be related to perceived criticism, where more negative interactions—including more negative nonverbal affect and more criticism—were observed by relatives who were rated as highly critical^82^. Hence, future studies can look to investigate individual differences in perceived sexist experiences when studying responses towards sexist events and possible interactions with other perpetrator characteristics.

On the other hand, the levels of upset experienced decreased as PC ratings for romantic partners increased only for female participants with high perceived sexism. We suggest that this may be due to differences in the expectations of behaviour from men with whom women share different relationships with, where they may be more “accepting” or “dismissive” of sexist behavior and/or attitudes in particular relational contexts. As mentioned above, Riemer et al.^35^ found that comments made by romantic partners (i.e. boyfriends) were always rated as less sexist than those made by bosses or strangers. Women are also more likely to accept sexist restrictions made by husbands compared to a co-worker^36^ and prescribe benevolently sexist behaviors for a romantic partner than a co-worker^66^. The present findings are indicative of an alignment with past findings suggesting that (certain) sexist behaviors and/or attitudes may be more “tolerated” or “disregarded” in romantic relationships where social interactions in these relationships are viewed less objectively. By doing so, women can maintain a favorable image of their partner in order to promote the maintenance and satisfaction of the relationship^76^. It is possible that female participants with high perceived sexism who also perceive their partners as highly critical of them are more likely to “disregard” or have a reduced emotional response towards such sexist-related criticism from their partner as they are able to identify and attribute the comments to their partner’s sexist beliefs as opposed to internalizing it. Female participants with high perceived sexism who also perceive their partner as highly critical of them may also be more “accustomed” to receiving criticism from their partner. This is consistent with findings that less intense feelings may arise when individuals perceive themselves to be the recipient of frequent and intentional hurt by their relational partners^83^. Another possible explanation is the motivation of romantic partners to share congruent beliefs over time^84^. As disapproval of female prowess from boys can create conflicting motives for females^85^, females may try to downplay their gender egalitarian beliefs to match that of their partners’, reducing the cognitive inconsistency and discomfort that arises from their initial preconceived egalitarian gender beliefs and perceptions of sexism. In romantic relationships, attitude alignment between partners tends to occur where it has been proposed that alignment with another’s attitudes is strongest when (i) motivation for relationship maintenance is present and (ii) the attitudes are relevant to the relationship^86^. For example, significant shifts in the direction of a partner’s attitudes were observed after discussion^87^. In the context of sexist attitudes, Kalmjin^84^ found that there was a positive effect of a partner’s sex-role attitudes on an individual’s later attitudes and Hammond et al.^88^ found that women with the perception that their male partner strongly endorsed benevolent sexism had greater and more stable benevolent sexist attitudes over time. Thus, they may become less emotionally aroused or are able to better regulate their emotions, and do not experience as much upset when they are confronted with criticism with sexist undertones.

### Limitations and future directions

There are several limitations to this current study. Firstly, the majority of participants that constitute this sample are undergraduate students. Although most of them have had some working experience, it is likely that most of these undergraduate students have not formally entered the workforce and only worked on a short-term basis. Thus, they might not have cultivated a deep relationship with their supervisors. This could have caused them to be less affected, and in turn experience less upset when receiving negative evaluative feedback or sexist comments from supervisors. Further studies could examine participants who have had accumulated formal working experiences to assess if there are similar effects of relationship type and perceived criticism on the level of upset experienced as seen in this study.

Secondly, the sexist-related criticism depicted in the vignettes in the present study were based on hostile sexism, which have been found to be more readily detected than those based on benevolent sexism. However, benevolent sexist experiences are more ambiguous but may also be construed as critical. Hostile and benevolent sexism also appear to be endorsed to different extents according to the relationship that they occur in (e.g.,^66^). Hence, future studies can look into comparing the influence of perceived sexism and relationship type on hostile versus benevolent sexism comments.

Thirdly, although the results in the study demonstrated how perceived criticism and perceived sexism moderated the ratings of upset in the female group towards sexist-related criticism, the study did not investigate the underlying mechanisms for these relationships. More research is required to disentangle these mechanisms for the effects observed in this study. As proposed in the discussion earlier, future studies can investigate possible differences in attributions made by female participants in relation to sexist-related criticism received from different sources to determine whether these attributions mediate the relationship between perceptions of criticism and sexism and the emotional response. In addition, future studies can also make use of neuroimaging experimental paradigms to investigate the relationships between perceptions of criticism and sexism with brain activity patterns relating to emotional reactivity and emotional processing to provide evidence on the sensitization or attenuation of the emotional response towards sexist-related criticism.

Finally, the study was conducted in the Singapore context, a largely collectivistic culture, and results may differ in other cultures. There are consistent findings of cross-cultural differences in emotional arousal levels. Individuals from Western cultures usually experience higher arousal emotions, such as anger, hostility and irritation, while those from Eastern cultures experience lower arousal emotions, including being calm, unaroused and sad^89^. There are also prominent cultural differences in rules of emotional display^90^. Since collectivistic cultures favor behavior that are harmonious with the group^91^ and postulate that individual notoriety may disrupt the collective identity of the culture^92,93^, reported perceptions and reactions to negative criticism may be less overtly pronounced as individuals from collectivistic cultures are socialized to avoid outward expressions of unhappiness or upset. Members of the collectivistic culture are expected to attune to and obey figures of authority or those who occupy higher positions in the social hierarchy relative to themselves due to an emphasis on distinct social hierarchies^94^. Individuals may harbor expectations of receiving criticism from figures of higher authority, such as parents and supervisors, cushioning the emotional impact of criticism received. Hence, there might have been greater motivation to conceal the true intensity of emotions experienced by downplaying the ratings of their perceptions of criticism and sexism, as well as feelings of upset than they truly feel. The present study can be replicated to other countries, especially Western countries that adopt individualistic values to investigate possible differences in how people of different cultures perceive and respond to negative criticism and sexism from social others.

## Conclusion

In conclusion, the current study serves to fill the gaps in literature examining the relationships amongst perceived criticism, perceived sexism and source of criticism on upset experienced in response to criticism. The most striking takeaway from this study is how sexist experiences can contribute to one’s perceptions of sexist experiences, which in turn moderates the association between perceptions of criticism in a relationship and levels of upset experienced towards sexist-related criticism originating from romantic partners and workplace supervisors. In a time where overt sexism is frowned upon yet remains a significant undercurrent in modern societies, this research study is an effort to contribute to the literature on gender and criticism. The current study opens up a line of possibilities in future research that seeks to examine the converging effects of perceived sexism and perceived criticism on social interactions.

## Data availability

The dataset analysed in this study is available in the open access institutional data repository at the link: https://doi.org/10.21979/N9/APYGCF.
